# Predicting Clinical Events Based on Raw Text: From Bag-of-Words to Attention-Based Transformers

**DOI:** 10.3389/fdgth.2021.810260

**Published:** 2022-02-21

**Authors:** Dmitri Roussinov, Andrew Conkie, Andrew Patterson, Christopher Sainsbury

**Affiliations:** ^1^Department of Computer and Information Sciences, University of Strathclyde, Glasgow, United Kingdom; ^2^Red Star Consulting, Glasgow, United Kingdom; ^3^NHS Greater Glasgow and Clyde, Glasgow, United Kingdom

**Keywords:** discharge summaries, BERT, clinical event prediction, pre-trained language models, transformers, deep learning

## Abstract

Identifying which patients are at higher risks of dying or being re-admitted often happens to be resource- and life- saving, thus is a very important and challenging task for healthcare text analytics. While many successful approaches exist to predict such clinical events based on categorical and numerical variables, a large amount of health records exists in the format of raw text such as clinical notes or discharge summaries. However, the text-analytics models applied to free-form natural language found in those notes are lagging behind the break-throughs happening in the other domains and remain to be primarily based on older bag-of-words technologies. As a result, they rarely reach the accuracy level acceptable for the clinicians. In spite of their success in other domains, the superiority of deep neural approaches over classical bags of words for this task has not yet been convincingly demonstrated. Also, while some successful experiments have been reported, the most recent break-throughs due to the pre-trained language models have not yet made their ways into the medical domain. Using a publicly available healthcare dataset, we have explored several classification models to predict patients' re-admission or a fatality based on their discharge summaries and established that 1) The performance of the neural models used in our experiments convincingly exceeds those based on bag-of-words by several percentage points as measured by the standard metrics. 2) This allows us to achieve the accuracy typically acceptable by the clinicians as of practical use (area under the ROC curve above 0.70) for the majority of our prediction targets. 3) While the pre-trained attention-based transformer performed only on par with the model that averages word embeddings when applied to full length discharge summaries, the transformer still handles shorter text segments substantially better, at times with the margin of 0.04 in the area under the ROC curve. Thus, our findings extend the success of pre-trained language models reported in other domains to the task of clinical event prediction, and likely to other text-classification tasks in the healthcare analytics domain. 4) We suggest several models to overcome the transformers' major drawback (their input size limitation), and confirm that this is crucial to achieve their top performance. Our modifications are domain agnostic, and thus can be applied in other applications where the text inputs exceed 200 words. 5) We have successfully demonstrated how non-text attributes (such as patient age, demographics, type of admission etc.) can be combined with text to gain additional improvements for several prediction targets. We include extensive ablation studies showing the impact of the training size, and highlighting the tradeoffs between the performance and the resources needed.

## 1. Introduction

Identification of patients who are likely to be readmitted or at higher risk of future complications can provide significant benefits for both patients and medical providers in terms of reducing heath risks, maintaining patients' quality of life and avoiding the markers of substandard health-care. The introduction of electronic health records (EHRs) and the move away from paper-based to almost entirely digital systems has led to the abundance of available electronic healthcare data and extensive patient histories. EHRs contain a wealth of information including patient demographics, laboratory test, prescriptions, radiological images, and clinical notes written by attending physicians. Compared to non-text (numerical, categorical etc.) attributes. the notes provide a richer picture of the patient since not only they list the symptoms and the treatment plans, but also describe more subtle aspects such as daily activities, patient mood and commitment to the regimens. At the same time, using the raw text for predicting major clinical events, such as fatality or re-admission, remains challenging due to the ambiguity and variety of human language. While machine-learning models, especially with deep (multi-layer) architectures, such as convolutional and recurrent networks, have lead to significant advances in natural language processing in the general domain (1), they have not been yet fully extended to healthcare applications. As we further elaborate in our review of related work, there is still no clear evidence that the deep learning approaches are more accurate than the classical “bags of words”, thus it is not surprising that the older techniques are still predominantly used by the practitioners.

Attention-based transformers (2), pre-trained on a large corpus to capture the language model (statistical distributions of words, phrases, sentences etc.), have been recently shown to be more effective than convolutional and recurrent neural models and advanced the state-of-the-art as measured by standard general benchmarks such as GLUE, MultiNLI, and SQuAD (3, 4). They have become de-facto standards for all modern text-analytics models in the general domain. At the same time, their use in healthcare remains limited, especially their application to the classification involving texts longer than a single paragraph, such as discharge summaries, since the transformers have an inherent input size limitation around a few hundred words.

Using a publicly available dataset with discharge summaries, we have adapted and compared several text classification models to predict readmission or a fatality at various time intervals and established that:

1) The performance of the deep neural models that we have tested exceeds those based on older but still currently dominant “bags of words” approaches by several percentage points. We believe that this finding is a major testament to the success of deep learning models, and particularly to the use of longer texts for clinical event predictions, which the prior work has not yet convincingly demonstrated.
2) The deep neural models allow us to achieve the accuracy above the “acceptable discriminative ability” (5) normally expected by the clinicians: for the standard metric of the area under the ROC curve, our models score at least 0.70 on the re-admission targets and 0.86 on the fatality-related targets. There is only a handful of prior reports of reaching that level of accuracy in the domain, and none of them is attributing the success to the use of raw-text.
3) The pre-trained attention-based transformer performs only on par with a simpler model that averages word embeddings when applied to full-length discharge summaries. However, the transformer still handles shorter text segments substantially better, which is demonstrated by the larger area under the ROC curve. Up to our knowledge, this is the first study confirming that the success of pre-trained language models in classifying text documents in the general domain extends to the healthcare, and specifically to the task of clinical event predictions based on discharge summaries (or any other medical texts of similar style and length)^1^.
4) In order to overcome the input size limit of the attention-based transformers, we have designed and tested several original modifications and show that without them, they would not reach the top performance. Those modifications are domain agnostic and thus can be used in other text classification applications as well.
5) Although non-text (numerical, categorical etc.) attributes are not the primary focus of our experiments presented here, we have also demonstrated how they can be successfully combined with our transformer-based model to provide additional performance gains.
6) We include ablation studies showing the impact of the training size, and demonstrating the tradeoffs between the performance and the computational resources needed.

The data that we used for our experiments can be obtained from a public source through a simple certification process^2^. We make our code publicly available^3^. The next section overviews the related works, followed by the description of the models used, empirical testing and conclusions.

## 2. Related Work and Models

### 2.1. Clinical Event Prediction

Automated classification of clinical texts such as cancer pathology reports and patient notes from hospital stays can potentially contribute toward health-related outcomes. For example, flagging specific cases can prevent patients from being discharged prematurely from Intensive Care Units (ICU). Additionally, preventable readmissions are associated with an increased risk of future complications and viewed as markers of substandard care (9).

While many domains have been revolutionized by an abundance of data, healthcare has been relatively slow in both the pace of research and the adoption of machine learning models to automatically analyse patients' records. Clinical text analytics approaches lag behind other domains, partly due to its risk-averse nature and to the legal challenges of releasing open research datasets (10, 11). Also, human annotation of EHRs can be extremely time-consuming and requires expensive expertise.

Another reason for text-analytics in healthcare falling behind other domains is that in many practical applications using **non-text** (numerical, categorical etc.) attributes works better than handling naturally ambiguous and diverse human language. While the models using non-text attributes have been enjoying some success they are also still progressing slowly: when (5) did a review of the approaches used at the time, they found out that predicting remained to be a challenging task and that the performance had not improved since a decade prior to that. While a single best performing model examined by the study scored 0.83 on the standard metric of the area under the ROC curve, very few models were able to achieve 0.70 which they designated as minimum “acceptable discriminative ability”. The approaches predominantly used categorical and numerical attributes such as various symptoms, diagnoses and patient test results, but not the raw text.

More recent works successfully applied deep neural models to make predictions based on other (non-text) variables as well: for example, Lin et al. (12) has built time-series models of EHR data for readmission prediction combining demographic data and ICD-9 disease codes. They used a pre-trained 300-dimension embedding vectors with recurrent (RNN) and convolutional (CNN) networks to achieve the area under ROC curve (AUC) of 0.791. Rajkomar et al. (13) offered a deep learning framework that combines text and non-text attributes. While reporting their model as successful, they still did not separately investigate the role of free-form text data in the overall performance, thus it remained unclear if using the text was actually helping.

### 2.2. Bag-of-Words vs. Deep Learning

The bag-of-words (BOW) models use only word counts to represent a text document. Thus, the word positions are ignored. Inspite of its simplicity, the approach often works well, and more sophisticated techniques don't always win by a substantial margin, especially in the challenging health-care domain. Walsh and Hripcsak (14) successfully applied BOW to the clinical notes for re-admission prediction. They pre-processed the text by using stemming and stopword removal and created vectors for each admission, combined with manually selecting the terms perceived to be of clinical significance. Using both the clinical text and selected features, they achieved the AUC of 0.68. Similar performance ranges were achieved in Curto et al. (9). Rumshisky et al. (15) applied topic analysis (Latent Dirichlet Allocation) on psychiatric notes to predict re-admission, which still also relied on bag-of-words.

Inspired by the success of deep learning in other domains, and using the same patient cohort as Walsh and Hripcsak (14), Jain et al. (16) used LSTM based networks with attention layers to build a prediction model for the 30-day readmission target. While their work showed that the application of attention layers can significantly boost the performance of LSTM-based recurrent networks over clinical text, their best performing model (AUC=0.71) was not significantly better than their bag-of-words baseline Logistic Regression model. Thus, their work still did not provide convincing evidence of the superiority of deep learning approaches over older techniques for this challenging task. We believe our current work provides much stronger evidence.

### 2.3. Pre-trained Transformers

Attention-based transformers (2) have proved to be very effective and become nowadays de-facto standards when implementing a language model (probability distribution over words, phrases, sentences, etc.). Once pre-trained on a very large corpus (e.g., Wikipedia or even larger-sized web crawls) the model is included as the main processing block within a classifier which is further “fine-tuned” (additionally trained) on a much smaller set of examples for a particular downstream application task [e.g., (3, 4)]. Instead of recurrent units with “memory gates" comprising the RNNs and process an input sequence in a certain direction, attention-based transformers use word positional embeddings and are more flexible and parallelizable than recurrent mechanisms.

To provide top performance, a transformer-based language model must be pre-trained on a text corpus that is from the same domain as the downstream application task. Therefore, clinical practitioners, who wish to apply those models, need to further pre-train publicly available general domain versions. Alsentzer et al. (8) additionally pre-trained a popular transformer-based language model [Bert, (3)] on Mimic-III text (17). They demonstrated success on several text-analytics tasks including inferencing, named entity recognition, de-identification, concept extraction and entity extraction, but not including classification of longer text such as clinical event detection based on discharge summaries that we consider here. We further fine-tune their publicly available model for those tasks in our experiments.

While doing this, we have to address a very serious limitation that the transformers have: since their algorithm includes iterating through all the pairs of its input, its input size has to be limited to 500–1,000 tokens, otherwise becoming computationally prohibitive. Since some words may be represented by several tokens, the limit approximately corresponds to a single paragraph. Our average discharge summary (from the same MIMIC-III) dataset is approximately 2,000 tokens, which is several times larger than the transformers' limit.

While several prior works [e.g., (18, 19)] modified transformer's architecture to be able to handle longer inputs, there are still no known pre-trained models of them for biomedical or clinical domains, most likely since pre-training transformer language models is a major computational burden (several weeks on current top GPUs or TPUs), and even more so for the models with the expanded input size limit.

There is also research on adapting publicly available pre-trained transformers for long document classification in the general domain by segmenting the input texts into shorter chunks. For example (20) explored applying this idea to IMDb reviews, Yelp reviews, Sogou News, and other similar datasets. The reported that the best overall classification accuracy is achieved by using only the first 128 and the last 382 tokens in each document. While we pursue somewhat similar segmentation strategy here, we do not confirm their observation for our discharge summaries: on the contrary, all the segments of the summaries turn out to be almost equally important.

## 3. The Models Explored

Here, we describe the models that we used for comparison, specifically: our implementation of bag-of-words model, our word embedding mean-pooling model and the attention-based transformer model. Our “Empirical Evaluation” section provides additional details on the implementations of convolutional and recurrent networks that we have used.

### 3.1. Bag-of-Words

The bag-of-words is a simple method that uses word counts to represent a text document. To implement it, we were guided by classical prior Information Retrieval approaches [e.g., (21)]. We only used words that occur in 10 documents or more. We did not apply any stopword removal, stemming or weighting. We simply treated those words as features in a linear classifier, trained as a logistic regression to minimize L2 loss between the predicted and actual labels. In our preliminary experiments we also tried other classification models including support vector machines, naive bayes, nearest neighbor, decision trees and random forests but obtained slightly worse results, thus our empirical section below reports only logistic regression. Preserving all the words, applying classical stemming and stopword removal did not result in any additional gains either, which is consistent with the observations in prior work, e.g., in Walsh and Hripcsa (14).

### 3.2. Mean-Pooling N-Gram Embeddings

We use one of the simplest deep neural models for text classification, which is inspired by Joulin et al. (22) (also known as “Fast-text” or “FastText”), but the model used here does not break words into sub-parts and does not use any additional text corpus to train the word embeddings. We made those decisions earlier in our experiments when we did not observe any impact of doing it. Thus, we use the implementation available in Gluon library library (see text footnote 2) without any modifications. The model assigns a trainable embeddings vector $\vec{v_{w}}$ to each word and n-gram (sequence of n words) *w* in the heuristically created vocabulary (preserving only the n-grams occurring enough many times). To classify a document, the mean-pooling is applied first to all the words and n-grams preserved in the document *d*: $\vec{v_{d}}=mean[\{(\vec{v_{w}}\}]$, and then $\vec{v_{d}}$ is fed to a fully-connected layer.

### 3.3. Attention-Based Transformer

**Figure 1:**
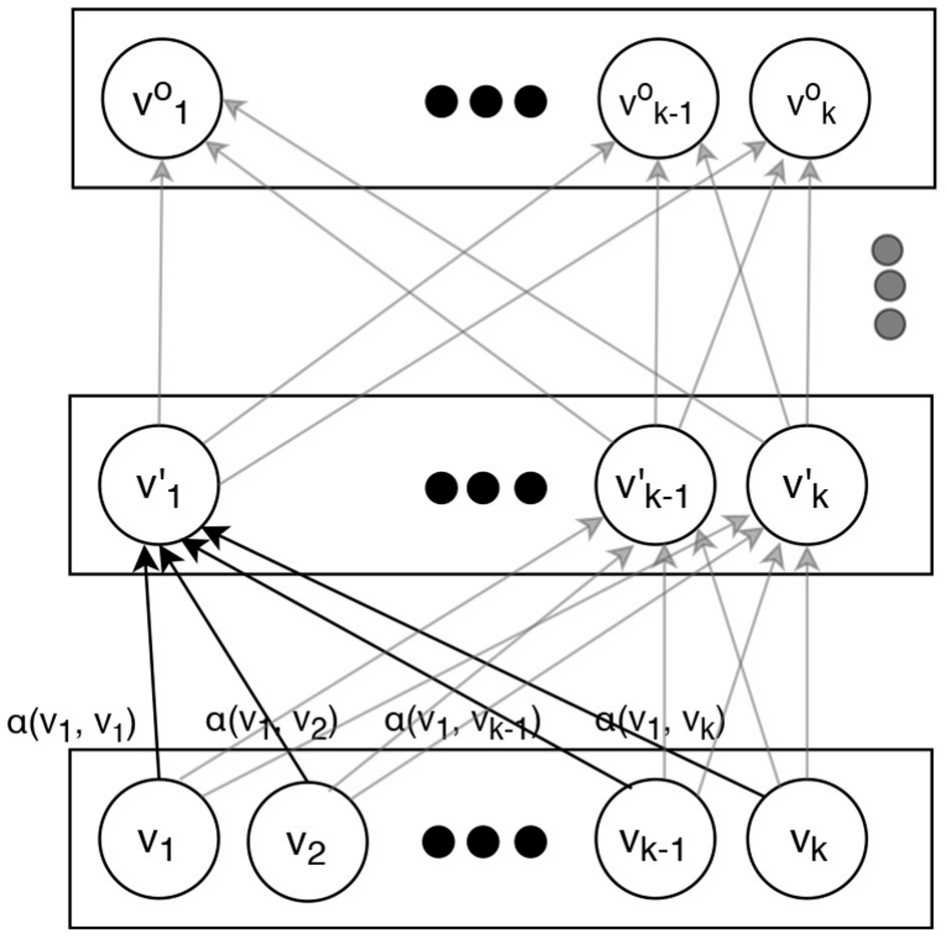
An attention-based transformer used in our experiments.

We only briefly overview the attention-based transformer model. For the details we refer to Vaswani et al. (2). An attention-based transformer is an *encoder* that can map a sequence of symbols (e.g., words) to a sequence of vectors. The diagram on Figure 1 illustrates how it operates. Instead of processing the input sequence in a certain direction as a recurrent network does, a transformer adds positional information (embedding) to the representation of each element in the sequence and then treats the elements uniformly regardless of their positions. The output sequence of vectors can be used in some downstream task, e.g., generating output words for machine translation (where attention-based transformers currently dominate) or a sentence classification such as sentiment analysis.

The conversion from inputs to the outputs is performed by several layers. Each layer applies the same transformations, varying only in their trained parameters. In order to obtain the vectors on a certain layer, the vectors from the layer immediately below are simply weighted and added together. After that, they are scaled and transformed by a linear and non-linear functions. For the latter, *tanh* is normally used:


(1) $$\begin{array}{r} {\vec{v_{i}}}^{\prime }=\tanh\left(W\cdot \overset{k}{\underset{t=1}{\sum }}\alpha_{t}\vec{v_{t}}\right) \end{array}$$

here, ${\vec{v_{i}}}^{\prime }$ is the vector in the *i*-th position on the upper layer, $\vec{v_{t}}$ is the vector in the *t*-th position on the lower layer, *W* is a trainable matrix (same regardless of *i* but different at different layers), and α_*t*_ is a trainable function of vectors $\vec{v_{i}}$ and $\vec{v_{t}}$, such that the weights for all $\vec{v_{t}}$ add up to 1. Normally, a scaled dot product of the vectors $\vec{v_{i}}$ and $\vec{v_{t}}$ is used:


(2) $$\begin{array}{r} \alpha_{t}=\vec{v_{i}}\cdot W^{\prime }\cdot \vec{v_{t}} \end{array}$$

where *W*′ is a trainable matrix (also same regardless of *i* and *t* at the same layer but different at different layers). The normalization to 1 is accomplished by using a *softmax* function.

This mechanism allows rich and contextual vector representations to be formed at the highest layers that can capture the entire content of a word sequence (e.g., a sentence) so it can be effectively used for any downstream application. Since here we are using it for text classification, as it is commonly done with attention-based transformers, we make our output classification decision based on the very first vector on the top level (sometimes informally called a “classification token”) ${\vec{v_{0}}}^{top}$, which is fed to a single layer fully connected neural network:


(3) $$\begin{array}{r} c=\mathrm{argmax}\left(W_{1}\cdot {\vec{v_{0}}}^{top}\right) \end{array}$$

where *c* is the output label (*true* or *false* in our case here) and *W*_1_ is a trainable matrix.

### 3.4. Resolving Transformer Input Size Limit

In spite of their great success, attention-based transformers have a serious limitation that needs to be considered. Their input sequence is normally limited in size. For example, the pre-trained model by Alsentzer et al. (8) that we use here (Clinical BERT) limits the input to 512 tokens. Since our application demands handling much larger inputs (our average discharge summary has approximately 2000 tokens), we segmented our text inputs by using non-overlapping sliding windows. As our empirical section illustrates, the choice of a proper model to connect those segments is crucial for the overall model to reach the top performance. Specifically, we have *designed and tested several architectural additions to the model to combine the segments, thus overcoming the imposed size limit*. Each of those models represents a certain trade-off between the flexibility of the model and computational demands:

1. LSTM CLS: The document classification token vector (${\vec{v_{0}}}^{top}$)_s_ from Vaswani et al. (2) of each segment *s* is fed in a sequence to a recurrent neural network (we used LSTM), which produces a vector of fixed size for the entire document:
  (4) $$\begin{array}{r} \vec{v_{d}}=\mathrm{LSTM}\left(\left\{{\left({\vec{v_{0}}}^{top}\right)}_{s}\right\}\right) \end{array}$$
  which in turn is used for making a classification decision by using a fully-connected network. The overall model (transformer + LSTM) is trained by back propagation.
2. LSTM on TOP LAYER: Instead of considering CLS token vectors only, this model feeds ALL the output vectors into LSTM:
  (5) $$\begin{array}{r} \vec{v_{d}}=\mathrm{LSTM}\left(\left\{{\left({\vec{v_{i}}}^{top}\right)}_{s}\right\}\right) \end{array}$$
  Since back-propagating that model is computationally prohibitive, the transformer is frozen, thus all its weights remain to be the same after initialization from the pre-trained values (as explained in the next section).
3. CONCATENATING TOP LAYER: same as (2), but instead of LSTM, all the top layer vectors are concatenated: $\vec{v_{d}}=\mathrm{concat}\left[{\left({\vec{v_{i}}}^{top}\right)}_{s}\right]$.
4. CONCAT CLS: All the CLS vectors are concatenated: $\vec{v_{d}}=\mathrm{concat}\left[{\left({\vec{v_{0}}}^{top}\right)}_{s}\right]$.
  The transformer is trainable, as in (1).
5. AVERAGE POOL CLS: Same as (4) but the vectors are averaged rather than concatenated: $\vec{v_{d}}=mean\left[{\left({\vec{v_{0}}}^{top}\right)}_{s}\right]$.
6. MIN POOL CLS: Same as (5), but minimum pooling applied instead: $\vec{v_{d}}=min\left[{\left({\vec{v_{0}}}^{top}\right)}_{s}\right]$.
7. MAX POOL CLS: Same as (5), but maximum pooling applied instead: $\vec{v_{d}}=max\left[{\left({\vec{v_{0}}}^{top}\right)}_{s}\right]$.

## 4. Empirical Evaluation

### 4.1. The Datasets

The MIMIC-III (17) consists of unstructured clinical notes as well as non-text attributes (such as patient age, demographics, type of admission etc.) from approximately sixty-thousand Intensive Care Unit (ICU) admissions into the intensive care unit at Beth Israel Deaconess Medical Center between 2001 and 2012. At the time of the study it was (and still is) the largest publicly available database comprising of de-identified health-related data.

Each admission is annotated by human experts with a set of ICD-9 codes that describe the diagnoses and the procedures. Each admission is also associated with a discharge summary which summarizes the information from the stay in a single document. An example of such is shown in Table 1. It can be seen that they often contain typos, specialized abbreviations and numbers, and can convey similar information in many different ways.

**Table 1:** A fragment of a discharge summary (an artificial example).

...Mrs Smith's overall left ventricular systolic function is normal. Her lungs are clear to auscultation bilaterally, coronary examination is regular rate and rhythm, abdomen is soft, nontender, nondistended. The patient's most recent laboratory values are from yesterday, which reveal a white blood cell count of 9. 1, hematocrit 29. 4, platelet count. She was placed under warming lights. On the evening of admission her temperature was again found to be low at 96.5, and she was again placed under lights. Given the recurrent nature for hypothermia she was brought to the nicu for evaluation. We have discharged Mrs Smith on regular oral Furosemide (40 mg OD) and we have requested an outpatient ultrasound of her renal tract which will be performed in the next few weeks. We will review Mrs Smith in the Cardiology Outpatient Clinic in 6 weeks time. After review from our social worker and occupational therapist, we have arranged a once-daily care package to assist Mrs Smith with her activities of daily living...

Patients can have more than one discharge summary for each admission, which is caused by a variety of reasons, including the fact that newer discharge summaries are merely addendums to older discharge summaries. To remove this multiplicity we concatenate multiple discharge summaries into a single text document while preserving the original sequence in the dataset. The average combined discharge summary in the MIMIC-III dataset is approximately 2,000 tokens when applying the tokeniser from the Clinical transformer that we used (8).

### 4.2. Prediction Targets

While there is no single standard benchmarking subset of MIMIC-III for clinical event predictions, so guided by prior works, we chose several targets related to a patient fatality or re-admission with the time intervals ranging from 7 to 365 days. Table 2 presents the overall statistics of each of the datasets. We balanced the training sets by randomly oversampling the minority class (the target) without replacement. The validation and test sets remained unbalanced.

**Table 2:** The statistics of the datasets for each prediction targets.

**Task**	**#negatives**	**#positives**
Re-admission within 7 days	44,961	1,109
Re-admission within 30 days	43,074	2,996
Re-admission within 90 days	41,183	4,887
Re-admission within 180 days	39,965	6,105
Re-admission within 365 days	38,692	7,378
Re-admission at any time in future	35,505	10,565
Fatality within 30 days	43,943	2,127
Fatality within 90 days	41,868	4,202
Fatality within 180 days	40,107	5,963
Fatality within 365 days	37,992	8,078

### 4.3. Metrics

As in most prior works, we use the area under the receiver ROC curve (AUC). It ranges from 0 (worst) to 1 (best) while random guessing resulting in 0.5. In clinical settings, only the values above 0.70 are considered useful, and those above 0.80 are considered good (5), with the exception of psychiatry where even lower performance is considered acceptable. We applied cross validation, each time using 75% of data for training, 10% as validation (development) set to choose our hyper-parameters and the remaining 15% to obtain the metrics to report.

### 4.4. Hyperparameters

For our Word Embedding Mean-Pooling model, we tested the embedding sizes in {50–1,000} range and the dropout rates in {0.1–0.5} range. For our RNN and CNN, we tested the embedding and context sizes in the same {50–1,000} range. Those ranges were typically used in similar applications. We used the same hyper-parameters for the transformer as in Devlin et al. (3), which allowed us to initialize our weights to those pre-trained in Alsentzer et al. (8) using medical texts^4^.

This configuration is reported below as “Transformer clinical.” We also tried initializing to the weights trained on general text from Devlin et al. (3). This configuration is reported as “Transformer general.” As traditional in machine learning, the result tables below present the best configuration as measured by the performance on the test set with the hyperparameters and stopping criteria maximizing the performance on the validation (development) set.

### 4.5. Implementation

Most training tasks have been accomplished on Tesla V100 GPU server with 16GB memory, where the most time-consuming complete configuration was taking approximately 15 min to train and 1 min to test. We trained each model for 5 epochs and chose the best model using validation (development) set. We report the metrics measured using the test set. Training, validation and test sets do not overlap.

Faster models were trained on GT2080 GPU processor with 8GB memory with the approximate speed of 10 min per epoch. The memory demand was dictated by the aggregate size of all the weights, which in the largest configuration was 0.5GB. The training algorithm makes additional memory allocations for caching/efficiency reasons (e.g., while holding all the 200Mb of training data in GPU memory speeds up the training, it is not essential for the model to operate). Our convolutional network is based on Zhang and Wallace (23). For our RNN, we used a GRU variation (24), which is known to work as well as LSTM while being slightly faster to train.

### 4.6. Results

Tables 3, 4 present the performance of the models that we have evaluated across multiple readmission and fatality prediction related tasks. The following can be observed:

1) Almost all our deep neural models achieve above *0.70* AUC, which is typically interpreted by clinicians as practically useful level of performance (5). Our most successful models reach 0.87 AUC. As we wrote in our Section 2, only a few prior works were achieving such levels, and none for the task of clinical event prediction based on discharge summaries. The deep learning scores are statistically different from the bag-of-words approach at the *0.01* level of significance. Thus, this work provides crucial evidence of superiority of deep learning over bag-of-words for this task and long text classification in healthcare domain in general, which the prior work is lacking.
2) While, mean-pooling of word embeddings is the winner among all our models, the differences between all the deep neural approaches are not statistically significant at the level of 0.01. Thus, using recently emerging pre-trained language model approach (Clinical BERT) did not result in detectable improvements in this experiment, so further experimentation was necessary, as described below.
3) Our attention-based transformer has achieved close to the best performance only when its underlying language model was trained on the medical domain, but not on the general domain and only after we introduced special architectural modifications to address its input size limit as we further elaborate below.

**Table 3:** The overall performance of the models on the entire discharge summaries for the re-admission prediction targets.

**Patient re-admission within:**	**Any time**	**7 days**	**30 days**	**90 days**	**180 days**	**a year**
**Bag-of-words model:**	0.761	0.673	0.713	0.751	0.757	0.763
**Deep Neural models:**						
Mean-Pooling Word Embeddings	0.787	**0.698**	**0.743**	**0.779**	**0.785**	0.791
Convolutional Neural Network	0.785	0.694	0.739	0.775	0.781	0.788
Recurrent Neural Network	0.786	0.696	0.738	0.777	0.783	0.790
Transformer General	0.778	0.688	0.731	0.768	0.774	0.780
**Transformer Clinical**	**0.788**	0.697	0.741	0.778	0.784	**0.793**

*The best values are in bold*.

**Table 4:** The overall performance of the models on the entire discharge summaries for the patient fatality prediction targets.

**Patient's fatality within:**	**30 days**	**90 days**	**180 days**	**a year**
**Bag-of-words model:**	0.845	0.832	0.838	0.844
**Deep Neural models:**				
Mean-Pooling Word Embeddings	0.875	0.862	0.867	**0.871**
Convolutional Neural Network	0.872	0.858	0.865	0.867
Recurrent Neural Network	0.875	0.861	0.867	0.873
Transformer General	0.864	0.851	0.857	0.861
**Transformer Clinical**	**0.876**	**0.863**	**0.868**	**0.871**

*The best values are in bold*.

Table 5 presents the comparison results of various attention-based transformer models to deal with its input size limitation to be able to process the entire discharge summaries, which are around 6 times longer in average than their 512 token limit. We present the percentage loss of the performance from the top model averaged across our targets. The following can be observed:

1. Applying proper segment combination mechanism such as considered by us here is crucial. Otherwise, the transformer-based approach would lag behind the mean-pooling model in performance.
2. The best performing segment combination model is LSTM CLS. Its difference from all the other models is statistically significant at the level of 0.01, except from Concat CLS and Mean-pool CLS models, which provide similar performance.
3. The models that frieze the transformer during training perform significantly worse, which shows that the word embeddings need to be changed from their initial values in order to reach the optimum. The fully-connected layer itself is not sufficiently powerful to learn how to transform the initial embeddings instead.
4. The models based on *max* or *min* pooling of CLS vectors perform significantly worse, which can be explained by the fact that pooling discards valuable information.

**Table 5:** Ablation: Average performance loss across all the targets relatively to the best combination model.

**Model**	**Relative AUC loss(%)**
**LSTM CLS**	**0**
LSTM on top layer	–9
Concat top layer	–12
Concat CLS	–2
Mean-pool CLS	–2
Min pool CLS	–21
Max pool CLS	–15

### 4.7. Discussion

We also experimented with shorter text segments, where the input size limitation of transformer-based models does not affect the results. We randomly chose 512-token sub-sequences from discharge summaries for the training, testing and validation sets accordingly. We used sliding non-overlapping windows and treated each portion as an independent data point, while still enforcing the train and test datasets not to share the segments from the same summaries. Tables 6, 7 present the performance of our deep neural models on those shorter portions. Those results support that on shorter segments, the transformer-based model always works at least as good as the mean-pooling model. It also works statistically significantly better in 3 out of 10 targets that we tried, and is never statistically significantly worse. The overall average relative AUC difference is around 0.005.

**Table 6:** Comparison of the deep neural models on a randomly chosen 512-token sub-sequence of discharge summaries for the re-admission prediction targets.

**Patient's re-admission within:**	**Any time**	**7 days**	**30 days**	**90 days**	**180 days**	**a year**
**Models:**						
Mean-Pooling Word Embeddings	**0.713**	0.630	0.678	**0.703**	0.709	0.714
CNN	0.707	0.627	0.673	0.697	0.711	0.712
RNN	0.708	0.625	0.675	0.695	0.703	0.711
Transformer Clinical	0.709	0.646^*^	**0.684**	0.697	**0.712**	**0.716**

* *Shows statistically significant difference from the second best result at the level of 0.01. The best values are in bold*.

**Table 7:** Comparison of the deep neural models on on a randomly chosen 512-token sub-sequence of discharge summaries for patient fatality prediction targets.

**Patient fatality within:**	**30 days**	**90 days**	**180 days**	**a year**
**Models:**				
Mean-Pooling Word Embeddings	0.791	0.779	0.785	0.780
CNN	0.787	0.773	0.781	0.778
RNN	0.789	0.774	0.786	0.776
Transformer Clinical	**0.808**	0.795^*^	**0.791**	0.788^*^

* *Shows statistically significant difference from the second best result at the level of 0.01. The best values are in bold*.

We also compared our deep neural models on the first 512-tokens (head) of the discharge summaries, according to the tokenizer from the Clinical transformer that we used, and also on the last 512-tokens (tail). The results in the Tables 8, 9 demonstrate a *strong superiority of the transformer-based model over mean-pooling*. On 7 out of 10 tasks, those differences are statistically significant, with the overall average AUC difference of 0.019. Considering that AUC ranges from 0.5 (random guessing) and 1.0 (perfect prediction, not currently achievable even by humans), the difference around 0.02 is practically important, especially if related to the lives and costs possibly saved. Similar results are shown in Tables 10, 11 when the models are compared on the head (first) segments only.

**Table 8:** Comparison of the deep neural models on the tail portions (last 512 tokens) of discharge summaries for the re-admission prediction targets.

**Patient's re-admission within:**	**Any time**	**7 days**	**30 days**	**90 days**	**180 days**	**a year**
**Models:**						
Mean-Pooling Word Embeddings	0.710	0.633	0.670	0.698	0.711	0.704
CNN	0.711	0.631	0.668	0.695	0.709	0.706
RNN	0.708	0.629	0.672	0.696	0.707	0.701
Transformer Clinical	0.745^*^	**0.639**	0.680^*^	0.725^*^	**0.727**	**0.739**

* *Shows statistically significant difference from the second best result at the level of 0.01. The best values are in bold*.

**Table 9:** Comparison of the deep neural models on the tail portions (last 512 tokens) of discharge summaries for patient fatality prediction targets.

**Patient's fatality within:**	**30 days**	**90 days**	**180 days**	**a year**
**Models:**				
Mean-Pooling Word Embeddings	0.782	0.780	0.782	0.805
CNN	0.778	0.778	0.780	0.806
RNN	0.781	0.777	0.779	0.801
Transformer Clinical	0.849^*^	0.829^*^	0.815^*^	0.822^*^

* *Shows statistically significant difference from the second best result at the level of 0.01. The best values are in bold*.

**Table 10:** Comparison of the deep neural models on the head (first 512 tokens) portions of discharge summaries for the re-admission prediction targets.

**Patient's re-admission within:**	**Any time**	**7 days**	**30 days**	**90 days**	**180 days**	**a year**
**Models:**						
Mean-Pooling Word Embeddings	0.746	0.646	0.694	0.707	0.726	0.737
CNN	0.747	0.642	0.692	0.703	0.727	0.735
RNN	0.744	0.644	0.692	0.704	0.722	0.734
Transformer Clinical	0.761^*^	0.654^*^	0.697^*^	0.737^*^	0.751^*^	0.759^*^

* *Shows statistically significant difference from the second best result at the level of 0.01. The best values are in bold*.

**Table 11:** Comparison of the deep neural models on the head (first 512 tokens) portions of discharge summaries for patient fatality prediction targets.

**Patient fatality within:**	**30 days**	**90 days**	**180 days**	**a year**
**Models:**				
Mean-Pooling Word Embeddings	0.783	0.788	0.768	0.802
CNN	0.781	0.784	0.762	0.799
RNN	0.780	0.785	0.764	0.798
Transformer Clinical	0.827^*^	0.826^*^	0.788^*^	0.841^*^

* *Shows statistically significant difference from the second best result at the level of 0.01. The best values are in bold*.

We find the results on the shorter portions of discharge summaries extremely encouraging! Combined with the previous paragraph reporting the results on the full summaries, they support our conclusion that when pre-trained on an appropriate corpora, transformer-based language models can be a powerful source of improvement against such competitive baseline as the word embedding mean-pooling model (Fast-Text), convolutional and recurrent networks. While some prior or works appearing in parallel to ours also reported similar observations, we believe this current work is the most convincing evidence at the moment. We have also used the strongest baselines for comparison, those rooted in deep-learning and known to work well for long document classification (Fast-Text, Convolutional and Recurrent Networks).

Our experiments have limitations as well. We have not considered interpretability of our results, leaving it for future work. One possibility is to evaluate the strength of all the word n-grams in the documents by removing them one-by-one during inference stage and looking for those that impact the classification the most way. Preserving only the most impactful n-grams and applying rule-based models to them may be another line of exploration that may result in an interpretable model.

We have only looked at a single database with one type of text entries (discharge summaries). We are leaving for future applying this to other EHR repositories and other types of text records (e.g., clinical notes). Our model can be also applied to other tasks such as diagnosis prediction, mortality risk estimation, or length-of-stay assessment.

We have only considered the simplest combination with non-text attributes such as patient demographics and admission types, leaving for future exploring the opportunities along this line including the use of ICD-9 disease codes. Numerical data can be also successfully integrated. Integration may be performed at deeper layer, e.g., using non-text attributes as input to the transformer's attention computation. Long term contextual information along the lines of scalable transformers [e.g., (18, 19)], can be also added that way. However, we have to keep in mind that any transformer-based model is computationally more expensive that simpler alternatives such as those considered here, so full adoption of then in health-care text analytics may still wait until standard laboratory capabilities sufficiently increase.

### 4.8. Ablation Studies

**Figure 2:**
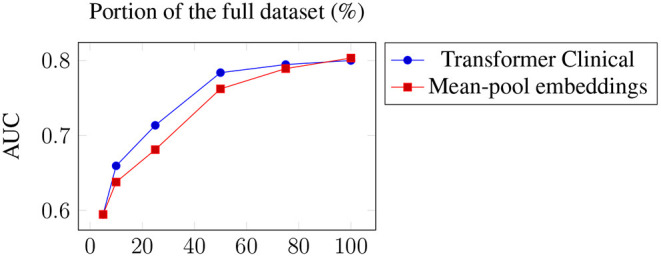
Average performance across the targets of the two best models when only a portion of the training data used.

We have also tested the impact of the training size on the model performance. Figure 2 presents the two best models, averaged across the targets, when only a certain portion of the training data used. The results suggest the importance of the dataset size and the possibility of further improvements when more training data is available. This is consistent with the observed positive correlation between the number of positive examples and the performance across the datasets. The transformer is the one more sensitive to the amount of training data, which is not surprising since it has a larger number of parameters to train.

Table 12 presents the average across the targets loss of the mean-pooling model performance for different values of maximum length of the n-grams used. Since large n results in larger vocabulary and embedding matrix, a trade off between the performance and the resources needed can be observed.

**Table 12:** Average performance loss across all the targets and the decrease in the resources required for various maximum n-gram lengths.

**Maximum n-gram length**	**Relative AUC loss (%)**	**Processing time reduction (%)**	**Allocated memory reduction (%)**
*n* = 3	0	0	0
*n* = 2	–1.1	–23	–33
*n* = 1	–3.2	–84	–61

**Figure 3:**
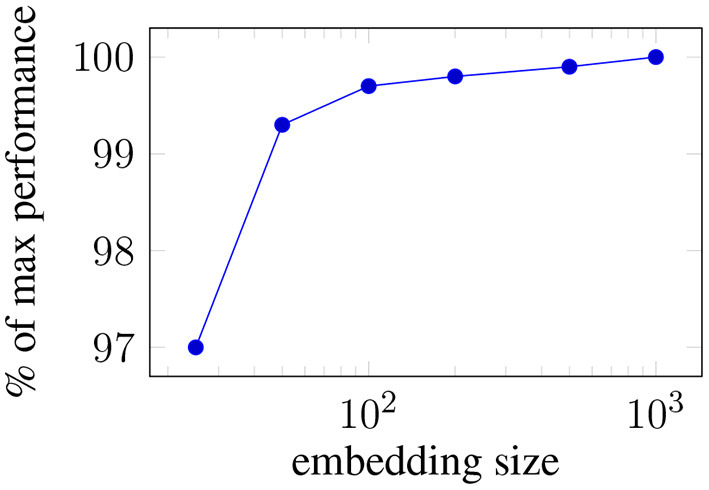
Ablating: Average performance loss across all the targets when the embeddings size is reduced.

Figure 3 presents the impact of the word embedding size on the average performance of the mean-pooling model.

### 4.9. Combining With Non-text Attributes

Although they were not the primary focus of our experiments presented here, as we noted in our related work section, various non-text attributes have been successfully used to predict clinical events. Here, we investigated a model that combines non-text and text attributes. Driven by the existing work, while still trying to preserve simplicity and replicability, we converted various available non-text attributes from the same Mimic III dataset into boolean representation and obtained 255 variables capturing such properties as patient age, gender, demographics, religion, type of admission (e. g. emergency), discharge location (home, hospital), type of insurance, but not including those describing medical treatments.

Tables 13, 14 present the performance of the fully-connected single-layer network that uses non-text attributes only. It also lists the performance of the combination of this model and our pre-trained transformer model. We simply concatenate the boolean vector representing the non-text attributes with transformer's CLS token. The following can be observed: 1) Non-text attributes provide practically useful classification performance (AUC greater than 0.70) on several targets, while still are below our free-text data models. This comparison may change if more elaborate non-text models are developed or additional attributed involved, but since non-text models were not the primary focus of our study, we left that for future research. 2) On 3 out of 10 targets, the combination adds at least 0.02 AUC to the performance of the transformer model, with the differences being statistically significant at the level of 0.01. Thus, these results support the conclusion that combining text and non-text data is promising.

**Table 13:** Combining the transformer-model with non-text attributes for the re-admission prediction targets.

**Patient's re-admission within:**	**Any time**	**7 days**	**30 days**	**90 days**	**180 days**	**a year**
**Models:**						
Non-text attributes	0.694	0.645	0.650	0.671	0.675	0.679
Transformer Clinical	0.788	0.697	0.741	0.778	0.784	0.793
Combination	0.808^*^	**0.704**	**0.742**	**0.783**	**0.786**	0.809^*^

* *Shows statistically significant difference from the second best result at the level of 0.01. The best values are in bold*.

**Table 14:** Combining the transformer-model with non-text attributes on the patient fatality prediction targets.

**Patient's fatality within:**	**30 days**	**90 days**	**180 days**	**a year**
**Models:**				
Non-text attributes	0.786	0.760	0.735	0.725
Transformer Clinical	**0.876**	**0.863**	0.868	0.871
Combination	**0.876**	0.860	0.883^*^	**0.875**

* *Shows statistically significant difference from the second best result at the level of 0.01. The best values are in bold*.

## 5. Conclusions

Using a publicly available database with Electronic Healthcare Records, we have explored several classification models to predict various clinical events (patient death or re-admission within certain time intervals) and established that 1) The performance of our models rooted in deep neural learning exceed those based on classical bag-of-words by several percentage points. To our knowledge, this is the first study that convincingly demonstrates superiority of deep learning over bag-of-words approaches for predicting clinical events based on raw text. 2) The deep neural models studied here achieve the accuracy typically acceptable by the clinicians as of practical use (area under the ROC curve 0.75 and above) thus their predictions can results in saving valuable resources and patients' lives. Prior work rarely achieved that level. 3) While for the full length discharge summaries, the model that averages the word embedding vectors worked on par with a pre-trained attention-based transformer, the latter performed significantly better on shorter portions of the summaries. This is consistent with the observations made in other domains and suggests that pre-trained language models will eventually win over healthcare text analytics as they have done so over other domains. While being complementary to the existing works, we believe our work is presenting the strongest so far evidence of this in terms of variety of targets, models compared and numerical improvements obtained. 4) Our original architectural additions to the attention-based transformer suggested in this study to overcome its input size limit are crucial to achieve top performance. Those modifications are domain agnostic and thus can be used in other text classification applications as well, which we are going to explore in future research, for example for document understanding tasks, question answering and information retrieval, which also often has to handle larger documents. 5) We have also successfully demonstrated how non-text attributes can be combined with text to gain additional improvements for some tasks. We are not aware of any past works combining transformers and non-text inputs for clinical event prediction. 6) We have performed extensive ablation studies showing the impact of the training size and evaluated our models implemented in simplified configurations which are less resource-intensive.

Thus, the results of our work can be directly applied by medical practitioners, e.g., by flagging specific cases as of being of higher risks for future complications, so deserving additional consideration before a discharge. While the physicians may not have time to review all the patient history when making important decisions, a trained algorithm can do that in a fraction of a second.

## Data Availability Statement

Publicly available datasets were analyzed in this study. This data can be found here: https://mimic.mit.edu/.

## Author Contributions

DR: leading, corresponding, and preparing manuscript. AC: preparing data and baseline tests. AP: implementing and testing the models. CS: advising on the data and models. All authors contributed to the article and approved the submitted version.

## Conflict of Interest

AC was employed by the company Red Star Consulting. The remaining authors declare that the research was conducted in the absence of any commercial or financial relationships that could be construed as a potential conflict of interest.

## Publisher's Note

All claims expressed in this article are solely those of the authors and do not necessarily represent those of their affiliated organizations, or those of the publisher, the editors and the reviewers. Any product that may be evaluated in this article, or claim that may be made by its manufacturer, is not guaranteed or endorsed by the publisher.

## Footnotes

^1^Our preliminary results were presented at HealTac 2020 and 2021 conferences. Also, somewhat parallel to our study, Gao et al. (6) reported text-classification experiments with a different pre-trained language model and different prediction targets from ours. Although they did not report any evidence of the superiority of the language model based approach, our works still complement each other. Another complementary worth mentioning work is Huang et al. (7), which tried to overcome transformer input size limit for 30-day re-admission task on the same dataset, but unlike us, pursued simple hueristic rather than deep-learning solutions and reported smaller improvements. Unfortunately, we were not able to use their pre-trained language model and are not aware of any follow up study that did. Thus, early in our experiments, we settled on using (8), which indeed later was used in numerous studies and became part of popular HuggingFace library.

^2^ https://mimic.physionet.org

^3^The URL will be provided in the camera-ready version.

^4^During the later stages of our project, more language models pre-trained on medical/clinical corpora became available through https://huggingface.co/. We experimented and informally obtained very similar results with BioBERT and BlueBERT but chose not run full set of experiments reported here due to the time and resource constraints.
